# Activating α7nAChR ameliorates abdominal aortic aneurysm through inhibiting pyroptosis mediated by NLRP3 inflammasome

**DOI:** 10.1038/s41401-022-00876-9

**Published:** 2022-02-25

**Authors:** Hui Fu, Qi-rui Shen, Yi Zhao, Min Ni, Can-can Zhou, Ji-kuai Chen, Chen Chi, Dong-jie Li, Guang Liang, Fu-ming Shen

**Affiliations:** 1 Department of Pharmacy, Shanghai Tenth People’s Hospital, Tongji University School of Medicine, Shanghai, 200072 China; 2 Chemical Biology Research Center, School of Pharmaceutical Sciences, Wenzhou Medical University, Wenzhou, 325035 China; 3 Department of Health Toxicology, Faculty of Naval Medicine, Second Military Medical University/Naval Medical University, Shanghai, 200433 China; 4 Institute of Nuclear Medicine, Tongji University School of Medicine, Shanghai, 200092 China

**Keywords:** abdominal aortic aneurysm, α7nAChR, inflammation, cell pyroptosis, NLRP3 inflammasome

## Abstract

Abdominal aortic aneurysm (AAA) is defined as a dilated aorta in diameter at least 1.5 times of a normal aorta. Our previous studies found that activating α7 nicotinic acetylcholine receptor (α7nAChR) had a protective effect on vascular injury. This work was to investigate whether activating α7nAChR could influence AAA formation and explore its mechanisms. AAA models were established by angiotensin II (Ang II) infusion in ApoE^−^^/−^ mice or in wild type and α7nAChR^−/−^ mice. In vitro mouse aortic smooth muscle (MOVAS) cells were treated with tumor necrosis factor-α (TNF-α). PNU-282987 was chosen to activate α7nAChR. We found that cell pyroptosis effector GSDMD and NLRP3 inflammasome were activated in abdominal aorta, and inflammatory cytokines in serum were elevated in AAA models of ApoE^−/−^ mice. Activating α7nAChR reduced maximal aortic diameters, preserved elastin integrity and decreased inflammatory responses in ApoE^−/−^ mice with Ang II infusion. While α7nAChR^−/−^ mice led to aggravated aortic injury and increased inflammatory cytokines with Ang II infusion when compared with wild type. Moreover, activating α7nAChR inhibited NLRP3/caspase-1/GSDMD pathway in AAA model of ApoE^−/−^ mice, while α7nAChR deficiency promoted this pathway. In vitro, N-acetylcysteine (NAC) inhibited NLRP3 inflammasome activation and NLRP3 knockdown reduced GSDMD expression, in MOVAS cells treated with TNF-α. Furthermore, activating α7nAChR inhibited oxidative stress, reduced NLRP3/GSDMD expression, and decreased cell pyroptosis in MOVAS cells with TNF-α. In conclusion, our study found that activating α7nAChR retarded AAA through inhibiting pyroptosis mediated by NLRP3 inflammasome. These suggested that α7nAChR would be a potential pharmacological target for AAA.

## Introduction

Abdominal aortic aneurysm (AAA) is one kind of chronic inflammatory disease with a focal dilation more than 50% of normal diameter in abdominal aorta, which may lead to vascular rupture accompanied by a severe morbidity and mortality [1]. Globally, about 150,000–200,000 annual deaths are caused by AAA [2, 3]. The incidence of AAA is up to 8% of men more than 65 years [4], which gradually increases as time goes on [5]. It is noteworthy that smoking, family history, hypertension and atherosclerosis also contribute to AAA formation. Endovascular aortic repair is currently the major treatment for patients with a large AAA (>50 mm), but there are still no effective drugs or therapies for asymptomatic and small AAAs [6]. Although more and more researches have been focused on AAA, the pathophysiological processes of AAA are still not fully understood. Therefore, exploration of possible molecular mechanisms for AAA disease is imperative for potential pharmacologic targets and effective strategies.

α7 nicotinic acetylcholine receptor (α7nAChR), which belongs to ligand-gated ion channels, is composed of five α7 subunits and involved in an unconventional anti-inflammation manner named as “cholinergic anti-inflammatory pathway” [7, 8]. Growing evidence showed that, activating α7nAChR could reduce inflammatory responses in many pathophysiological processes, such as Alzheimer’s disease, multiple sclerosis and ischemia/reperfusion injury [9–11]. Our previous studies found that, activation of α7nAChR could inhibit neointima formation following vascular injury through alleviating the productions of oxidative stress and arterial inflammation [12]. It was certain that the adventitial and medial inflammation played an important role in the development of AAA by regulating the productions of pro-inflammatory cytokines and reactive oxygen species (ROS) [13]. However, whether α7nAChR mediated anti-inflammatory effects play a role in the AAA development and its possible mechanisms, are still largely unknown.

Cell pyroptosis, which is induced by endogenous danger molecules or pathogen infection, could trigger the release of numerous inflammatory cytokines, especially IL-1β and IL-18 [14]. Cell pyroptosis is mainly mediated by inflammatory caspases, such as caspase-1 or caspase-11 (in mice) as well as caspase-4 or caspase-5 (in human) [15]. With the discovery of cell pyroptosis executor gasdermin D (GSDMD), cleavage of GSDMD was considered as a pivotal event to initiate cell pyroptosis [16–18]. Nod-like receptor family pyrin domain-containing 3 inflammasome (NLRP3 inflammasome, also named as NALP3 inflammasome), as one of the best-studied inflammasome complexes, could cleave GSDMD and induce cell pyroptosis after gastrointestinal norovirus infection [19]. Recently, Deng et al. found that targeting α7nAChR could inhibit the activation of NLRP3 inflammasome to prevent inflammatory responses in pulmonary hypertension [20]. In addition, a potential NLRP3 inflammasome inhibitor MCC950, had an protective effect on preventing aortic destruction and aneurysm in mice [21]. At present, whether α7nAChR-mediated inhibition of NLRP3 inflammasome and cell pyroptosis was involved in the AAA progression, has not been put forward.

In the present study, AAA models were induced by Ang II infusion in ApoE^−/−^ mice, or in wild type (WT) and α7nAChR^−/−^ mice. In vitro, mouse aortic smooth muscle (MOVAS) cells were treated with tumor necrosis factor-α (TNF-α). PNU-282987 was chosen to activate α7nAChR in vivo and in vitro. We hypothesized that activating α7nAChR was able to retard AAA formation via inhibiting pyroptosis by dampening NLRP3 inflammasome activation.

## Materials and methods

### Animals and AAA models

Male ApoE^−^^/−^ mice were purchased from Charles River in Beijing. Age-matched male α7nAChR^−/−^ mice (Stock number: 003232) and WT mice were purchased from Jackson Laboratory in USA, and bred in our animal facility. All mice were housed under a relative humidity about 50%, constant temperature (22–25 °C), standard light conditions (12 h-light/12 h-dark cycle) and were free to food and water. The whole experimental procedures were approved by the Institutional Animal Care and Use Committee of Tongji University and in accordance with the Guide for the Care and Use of Laboratory Animals, which was published by the Ministry of Health, People’s Republic of China and National Institutes of Health.

Male ApoE^−/−^ mice (about 6-month-old), male WT and α7nAChR^−/−^ mice (about 12-month-old) were used to induce AAA formation by Ang II. Briefly, mouse was anesthetized with pentobarbital sodium (50 mg/kg, ip) and implanted with an osmotic minipump (#2004#, Durect corporation, USA), which could deliver Ang II at a rate of 1000 ng·kg^−1^·min^−1^ for 4 week successively. PNU-282987 (1 mg·kg^−1^·d^−1^, ip) was injected to activate α7nAChR in ApoE^−/−^ mice. Four weeks after surgery, mice were killed after anesthesia to obtain the abdominal aorta for analysis. An aneurysm was defined by at least 50% dilation in the diameter of abdominal aorta from Ang II-infused mice as compared with the control.

### Morphological analysis and immunohistochemistry staining in mice

Mice were killed and the abdominal aortas were harvested for morphological analysis. To accurately measure the aortic diameter, the periadventitial tissue was carefully removed from aortic wall under a dissecting microscope. The aorta was then embedded in paraffin and cut into 4 µm for hematoxylin & eosin (H&E) staining. The external diameter of abdominal aorta was measured with an electronic caliper, combined with a software (Image J) after H&E staining. Elastin integrity of the aortic wall was evaluated by Verhoeff-van Gieson (VVG) staining and graded as four levels according to the reference [22]: (1) score 1, the elastic laminae was intact and with no degradation; (2) score 2, there were some breaks in the elastic laminae; (3) score 3, severe elastin fragmentations or loss in aorta; (4) score 4, severe elastin degradation occurred in reputed aortic sites.

For immunohistochemical (IHC) staining, the paraffin-embedded sections of AAA tissues were incubated with primary antibodies at 4 °C overnight. After washing, the secondary antibodies were used for 1 h. Then cell nuclear was stained with DAPI after washing. All images were taken by an inverted microscope and the inflammatory responses were assessed. Primary antibodies of TNF-α (BA0131, Boster, China), IL-1β (ab205924, abcam, UK) and IL-18 (ab71495, abcam, UK), were used for IHC staining.

### Measurement of inflammatory cytokines in serum

After 4 week Ang II or saline infusion, mice were under anesthetic to euthanasia with an overdose of pentobarbital sodium (150 mg/kg) by peritoneal injection. Blood samples were collected and used to detect inflammatory cytokines (TNF-α, IL-1β, IL-6 and IL-18) by enzyme-linked immunosorbent assay. Briefly, 100 µL sample or standard together with the antibody solution was added into the corresponding wells in the plate, which was then placed at 37 °C for 90 min. After washing the plate, ABC or TMB solution was added into the plate. The absorbance at 450 nm was detected by a Tecan infinite M200 reader (Tecan, Swiss) at 10 min after the stop solution was added, and the concentration of the inflammatory cytokines in serum was calculated.

### RT-PCR

In brief, total RNA was extracted from the aortas in ApoE^−/−^ mice with Trizol reagent (Takara, China). The RNA was reversed and followed by real-time PCR analysis (Bimake, USA). The mRNA level of the inflammatory cytokine was normalized by GAPDH.

### Cell culture and treatments

Mouse aortic smooth muscle cells (MOVAS) were purchased from American Type Culture Collection and cultured in high-glucose DMEM supplemented with 1% penicillin-streptomycin and 10% fetal bovine serum. Different concentrations of ROS scavenger *N*-acetylcysteine (NAC) were used to detect their effects on the cell viability in MOVAS. In total, 2 mmol/L of NAC was selected in the following experiment. In addition, MOVAS were pretreated with PNU-282987 (10 µmol/L) to activate α7nAChR for 1 h, and then stimulated with TNF-α (100 ng/mL) for 24 h. Subsequently, cells were collected for the further detection.

### The siRNA mediated NLRP3 knockdown in MOVAS

The NLRP3 siRNA mediated knockdown in MOVAS was performed. siRNA for NLRP3 was obtained from GenePharma Corporation (Shanghai, China). The siRNA transfection reagent, Lipofectamine 2000, was used (Thermo Fisher Scientific Inc., USA). Briefly, MOVAS cells were plated in 6-well plates and allowed to reach an ~40%–50% confluence. NLRP3 siRNA in Opti-MEM medium mixed with Lipofectamine 2000, was added to MOVAS for 6 h according to the manufacturer’s protocol. The medium was replaced with normal medium and cells were incubated for another 24 h. Cells were then treated with TNF-α (100 ng/mL) for 24 h. Western blot analysis was performed to confirm downregulation of NLRP3.

### Detection of reactive oxygen species and hydrogen peroxide in MOVAS

A ROS kit (Beyotime, China) was used to detect ROS levels in MOVAS. Briefly, MOVAS were harvested after different treatments and incubated with 10 µmol/L dichlorodihydrofluorescein diacetate at 37 °C for 20 min. Then cells were washed with PBS at least three times and detected for fluorescence by flow cytometry (BD FACS Calibur^TM^ Flow Cytometer, USA).

The hydrogen peroxide (H_2_O_2_) production was detected by a H_2_O_2_ Assay Kit (Beyotime, China). MOVAS were seeded in a 6-well plate with different treatments for 24 h and were washed with PBS twice. In total, 200 µL sample preparation buffer was added in each well. Then cell lysis was collected and centrifuged at 12,000 × *g* for 5 min at 4 °C. Cell samples were added into a 96-well plate and incubated with different work solutions according to the protocol at room temperature for 30 min. Finally, the absorbance was gained at 562 nm by a Tecan infinite M200 reader (Tecan, Swiss) for H_2_O_2_ production. The statistical results were normalized against the control group.

### Measurement of mitochondrial DNA and lactate dehydrogenase in MOVAS

The relative of mitochondrial DNA (mtDNA) was measured according to Chen’s work which has been recently published [23]. Briefly, MOVAS were treated with different treatments and obtained for DNA with a DNA Extraction Kit (D0063, Beyotime, China). Nuclear control and mtDNA primers were designed to detect the relative mtDNA copy number with RT-PCR assay.

Lactate dehydrogenase (LDH) release was detected with an LDH Assay Kit (Beyotime, China). MOVAS were seeded in a 48-well plate with different treatments for 24 h. The 48-well plate was centrifuged at 400 × *g* for 5 min to obtain cell supernatants. The cell supernatant (120 μL/well) was added into a new 96-well plate and incubated with 60 μL/well LDH detection working solution at room temperature. The plate was kept in a dark place for 30 min and the absorbance was measured with a microplate reader at 490 nm (Tecan, Swiss).

### Cell morphology by scanning electron microscope

MOVAS cells were cultured on the coverslips in 6-well plate and treated by TNF-α with or without PNU-282987. Cells were washed gently with PBS twice and fixed with 2.5% glutaraldehyde for 2 h at room temperature. After the fixation, samples were dehydrated and coated with gold-palladium. Cell morphology was examined subsequently with a scanning electron microscope (SEM, Hitachi-SU8200, Japan).

### Western blot

Mouse aortic samples and MOVAS cell extracts were quantitated by a BCA kit (Thermo Scientific, USA) and subjected to Western blot measurement. The primary antibodies incubation was carried out at 4 °C overnight, followed by secondary antibodies at room temperature for 1 h. The protein expression was detected by Odyssey infrared imaging system (LICOR, USA) and analyzed by Quantity One software. The protein expression was normalized to corresponding GAPDH or Tubulin expression. In our work, the primary antibodies of GSDMD (ab209845), Caspase-11 (ab180673) and α7nAChR (ab10096), were purchased from abcam (UK). The primary antibodies of NLRP3 (#15101), ASC (#67824), Bcl-2 (#2870) and IL-1β (#31202) were purchased from Cell Signaling Technology (USA). Cleaved Capsase 1 (sc-56036) and OPN (Osteopontin, sc-21742) antibodies were obtained from Santa Cruz (USA). α-SMA (14395-1-AP) and IL-18 (10663-1-AP) antibodies were purchased from proteintech (USA). Tubulin (AF0001), Bax (AF0054) and GAPDH (AG019) antibodies from Beyotime (China) were used.

### Statistical analysis

The data in violin plots were shown as each value per group. Other data were shown as means ± SD, and the data were normalized with its matched group. The survival curve was analyzed with Log-rank (Mantel–Cox) test. One-way ANOVA for three or four groups and unpaired *t*-test for two groups were used for statistical analysis. Fisher’s exact test was used for AAA incidence analysis. A value of *P* < 0.05 was regarded as statistical significance. All the statistical analyses were performed by GraphPad Software 8.0.

## Results

### Cell pyroptosis and NLRP3 inflammasome were involved in AAA formation

**Fig. 1:**
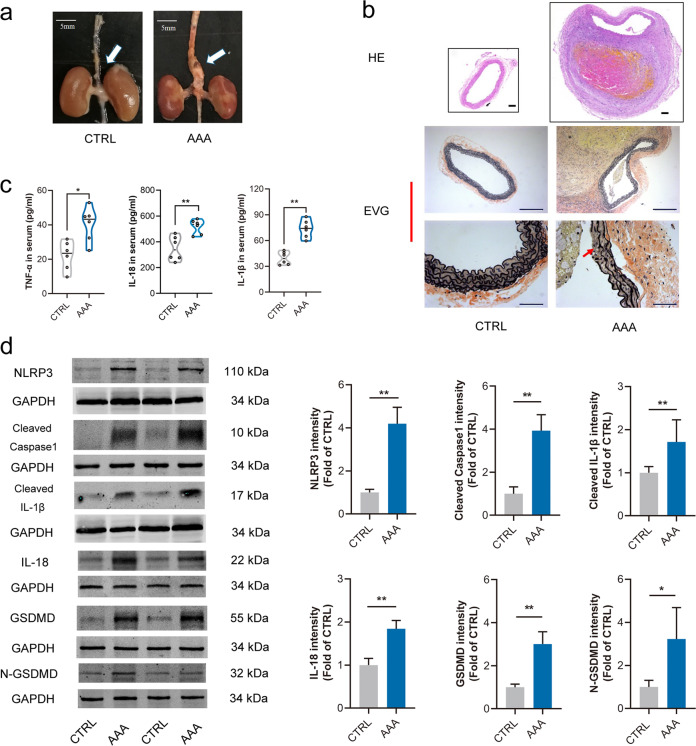
Cell pyroptosis and NLRP3 inflammasome were involved in AAA formation. Male ApoE^−/−^ mice were infused with angiotensin II (Ang II, 1000 ng·kg^−1^·min^−1^) for 4 week to induce abdominal aortic aneurysm (AAA), the control (CTRL) was infused with normal saline. **a** Representative images of abdominal aortas in ApoE^−/−^ mice, scale bar = 5 mm. **b** HE and EVG staining displayed intraluminal thrombus and disruption of the elastin in AAA mice, scale bar = 100 µm (up), or 200 µm (middle), or 50 µm (down). **c** Serum TNF-α, IL-18 and IL-1β were significantly increased in AAA mice, data were shown as each value of inflammatory cytokines. *n* = 6 mice per group, *P < 0.05, **P < 0.01 vs. CTRL. **d** Expressions of NLRP3, Cleaved caspase-1, Cleaved IL-1β, IL-18, GSDMD and N-GSDMD were increased in AAA tissues. *n* = 6 mice per group, data were shown as means ± SD. *P < 0.05, **P < 0.01 vs. CTRL.

ApoE^−/−^ mice were used to induce AAA formation by Ang II, which is regarded as the most wildly used AAA model in mice [24–26]. Morphologically, the aortas were obviously dilated in AAA mice as compared with the control (Fig. 1a). Intraluminal thrombus and disruption of the elastin were also found in AAA mice (Fig. 1b).

Cell pyroptosis, dependent on the caspase family, was characterized as the rupture of cell membrane and release of abundant pro-inflammatory cytokines [14], especially IL-1β and IL-18. NLRP3 inflammasome was considered to be an important activator of cell pyroptosis, and activation of NLRP3 inflammasome could produce a mass of IL-1β and IL-18 [27, 28]. It was found that NLRP3 inflammasome was activated as evidenced by the increased protein levels of NLRP3, Cleaved caspase-1, Cleaved IL-1β and IL-18 in abdominal aortas from AAA mice. Meanwhile, the expression of cell pyroptosis effector GSDMD was also increased (Fig. 1d). In addition, serum levels of TNF-α, IL-18 and IL-1β were increased in the AAA group (Fig. 1c). These together suggested that cell pyroptosis and NLRP3 inflammasome were involved in the pathogenesis of AAA in ApoE^−/−^ mice.

### Activating α7nAChR slowed down AAA formation

**Fig. 2:**
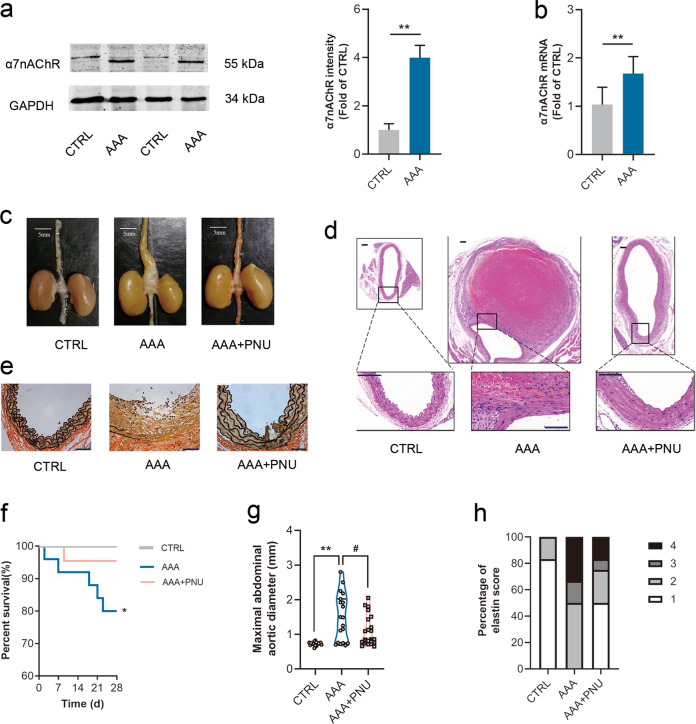
Activating α7nAChR slowed down AAA formation. Male ApoE^−/−^ mice were infused with Ang II to induce AAA. PNU-282987 (PNU) was injected to activate α7nAChR. **a**, **b** Both protein and mRNA levels of α7nAChR were increased in aortas from AAA mice. *n* = 5–7 mice per group, data were shown as means ± SD, **P < 0.01 vs. CTRL. **c** Representative images of abdominal aortas in ApoE^−/−^ mice, scale bar = 5 mm. **d** Representative images of HE staining in ApoE^−/−^ mice, scale bar = 100 µm (up) or 50 µm (down). **e** Representative images of EVG staining in ApoE^−/−^ mice. scale bar = 50 µm. **f** The survival curve in ApoE^−/−^ mice. *n* = 20 mice for CTRL group; *n* = 26 mice for AAA group; and *n* = 22 mice for AAA + PNU group. **g** PNU treatment reduced maximal abdominal aortic diameters with Ang II infusion in ApoE^−/−^ mice. *n* = 19–21 mice per group, data were shown as each value of per mouse. **P < 0.01 vs. CTRL; ^#^*P* < 0.05 vs. AAA. **h** PNU treatment improved the elastin integrity, *n* = 12 mice per group

Our previous study found that α7nAChR acted as a protective role in vascular injury [12]. In this work, it was found that the expression of α7nAChR was obviously increased in both protein and mRNA levels in AAA tissues of ApoE^−/−^ mice (Fig. 2a, b). To investigate whether activating α7nAChR could retard AAA formation, PNU-282987 (a selective α7nAChR agonist) was used. PNU-282987 treatment did not change aortic diameter or elastin integrity in ApoE^−/−^ mice (Fig. S1). However, PNU-282987 treatment significantly reduced maximal aortic diameters induced by Ang II infusion in ApoE^−/−^ mice (Fig. 2c, d, g), and increased the survival rate (Fig. 2f). In addition, activating α7nAChR could preserve the elastin integrity in abdominal aortas in Ang II-infused ApoE^−/−^ mice (Fig. 2e, h). These indicated that activating α7nAChR could slow down AAA expansion in ApoE^−/−^ mice.

### Activating α7nAChR inhibited inflammation and prevented the phenotype switch of VSMCs in AAA mice

**Fig. 3:**
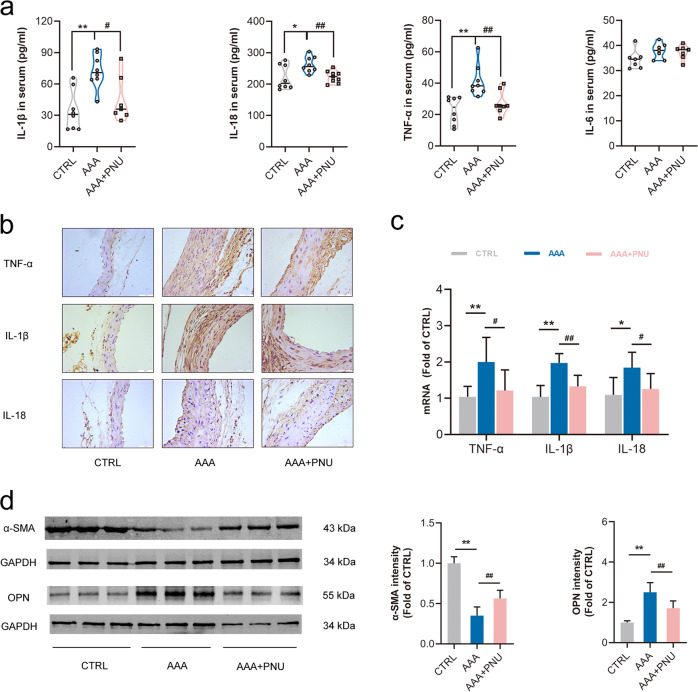
Activating α7nAChR inhibited inflammation and prevented the switch of VSMCs to a synthetic phenotype in AAA mice. Male ApoE^−/−^ mice were infused with Ang II to induce AAA. PNU-282987 (PNU) was injected to activate α7nAChR. **a** PNU inhibited inflammation in ApoE^−/−^ mice with Ang II. *n* = 7–8 mice per group, data were shown as each inflammatory cytokine’s concentration. *P < 0.05, **P < 0.01 vs. CTRL; ^#^*P* < 0.05, ^##^*P* < 0.01 vs. AAA. **b** Immunohistochemistry for TNF-α, IL-1β and IL-18 in aortas from ApoE^−/−^ mice, scale bar = 50 µm. **c** Real-time PCR for TNF-α IL-1β and IL-18 expression in aortas from ApoE^−/−^ mice. *n* = 6–7 mice per group, data were shown as means ± SD. *P < 0.05, **P < 0.01 vs. CTRL; ^#^*P* < 0.05, ^##^*P* < 0.01 vs. AAA. **d** Activating α7nAChR prevented the decrease of α-SMA (a contractile phenotype marker of VSMCs) and increase of OPN (a synthetic phenotype marker of VSMCs) with Ang II infusion. *n* = 6 mice per group, data were shown as means ± SD. **P < 0.01 vs. CTRL; ^##^*P* < 0.01 vs. AAA.

It was well-accepted that vascular inflammation promoted the development of AAA [5]. Therefore, inflammatory cytokines in both serum and abdominal aorta were detected. Compared with the control, inflammatory cytokines (IL-1β, IL-18 and TNF-α) in serum were increased in ApoE^−/−^ mice by Ang II infusion, but was notably inhibited with PNU-282987 treatment (Fig. 3a). Immunohistochemistry study and RT-PCR analysis confirmed that the increased expressions of TNF-α, IL-1β and IL-18 were significantly prevented by activating α7nAChR in AAA mice (Fig. 3b, c). These suggested that activating α7nAChR decreased inflammatory responses in AAA mice.

Vascular smooth muscle cells (VSMCs) had an important effect on maintaining the integrity of aortic wall. It has been reported that the VSMCs phenotype could be transformed from a contractile phenotype to a synthetic phenotype in the pathophysiological process of AAA [29]. Thus, the expressions of α-SMA (a marker of contractile phenotype) and OPN (a marker of synthetic phenotype) were examined to determine the role of α7nAChR on VSMCs phenotype in AAA. It was found that activating α7nAChR could partially prevent the decrease of α-SMA and the increase of OPN in AAA from ApoE^−/−^ mice (Fig. 3d). These results revealed that the protective effects of α7nAChR on AAA might involve preventing the switch of VSMCs from a contractile to synthetic phenotype.

### α7nAChR deficiency promoted AAA formation and inflammation

Given that activating α7nAChR displayed a protective role in AAA in our work, next we investigated whether α7nAChR deficiency would contribute to AAA formation. There were no obvious differences in body weight and ratio of tissue to body weight between age-matched WT and α7nAChR^−/−^ (KO) mice (Fig. S2).

**Fig. 4:**
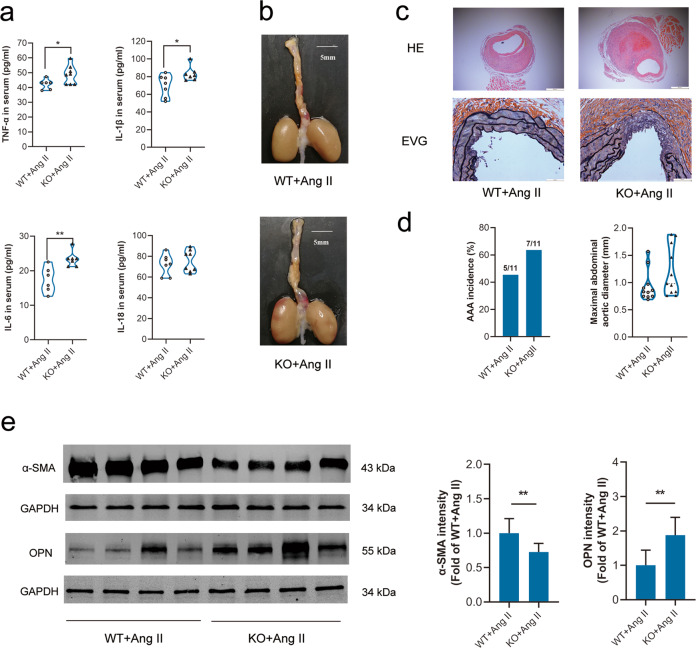
α7nAChR deficiency promoted AAA formation and inflammation. Age-matched male wild type (WT) and α7nAChR^−/−^ (KO) mice with Ang II infusion for 4 week to induce AAA. **a** α7nAChR deficiency increased inflammatory cytokine expression in serum. *n* = 6–8 mice per group, data were shown as each cytokine’s concentration, *P < 0.05, **P < 0.01 vs. WT + Ang II. **b**, **c** α7nAChR deficiency accelerated the abdominal aortic dilation and displayed more serious disruption of elastin, scale bar = 5 mm (**b**), 500 µm (**c** up) or 50 µm (**c** down). **d** The AAA incidence and the maximal abdominal aortic diameter in WT and α7nAChR^−/−^ mice infused with Ang II. *n* = 11 mice per group, the maximal abdominal aortic diameter was shown as each value. **e** VSMCs were more inclined to the synthetic phenotype in AAA tissues in α7nAChR^−/−^ mice with Ang II infusion. *n* = 8 mice per group, data were shown as means ± SD, **P < 0.01 vs. WT + Ang II.

It has been reported that aging could promote AAA formation with Ang II infusion [30]. Therefore, age-matched (12-month-old) WT and α7nAChR^−/−^ mice were used to induce AAA formation. α7nAChR deficiency increased the expressions of inflammatory cytokines (IL-1β, IL-6 and TNF-α) in serum, accelerated abdominal aortic dilation and displayed more serious disruption of elastin by Ang II infusion (Fig. 4a–c). It was also found that the AAA incidence of Ang II-infused WT mice (45.5%, that is 5/11) was lower than that of age-matched (about 12 months) Ang II-infused α7nAChR^−/−^ (63.6%, that is 7/11), and the average maximal abdominal aortic diameter in α7nAChR^−/−^ mice was longer than that of WT mice. Both the AAA incidence and the average maximal abdominal aortic diameter in α7nAChR^−/−^ mice displayed a trend of increasing (Fig. 4d). These indicated that α7nAChR deficiency promoted AAA formation and inflammation. In addition, compared with Ang II-infused WT mice, VSMCs were more inclined to the synthetic phenotype in abdominal aortas in α7nAChR^−/−^ (KO) mice with Ang II infusion (Fig. 4e).

### Activating α7nAChR decreased cell pyroptosis in AAA mice

**Fig. 5:**
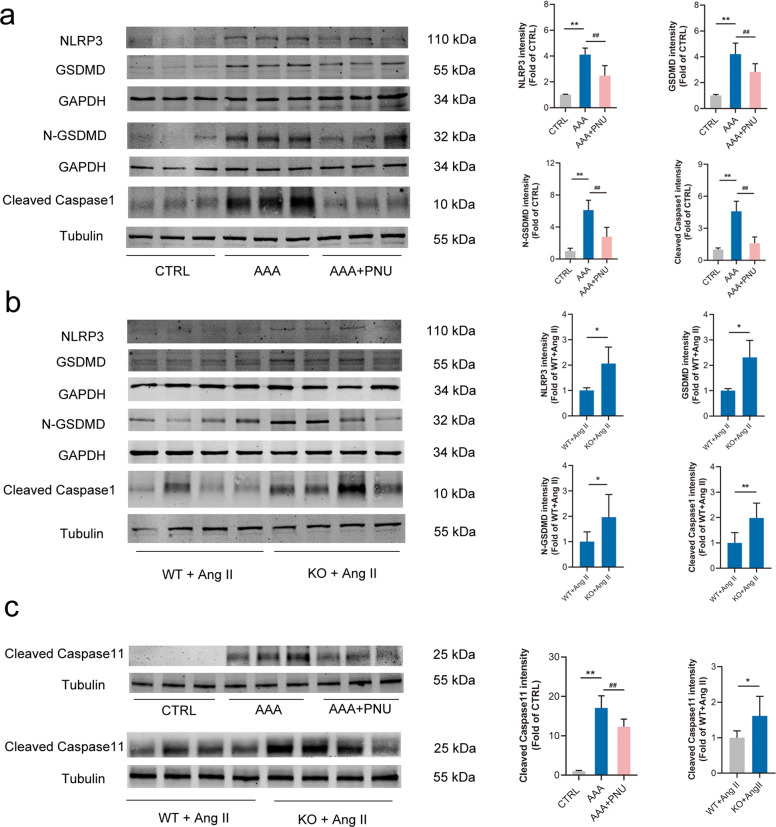
Activating α7nAChR inhibited cell pyroptosis in AAA mice. Male ApoE^−/−^ mice were infused with Ang II to induce AAA and PNU-282987 (PNU) was injected to activate α7nAChR in ApoE^−/−^ mice. Age-matched male wild type (WT) and α7nAChR^−/−^ (KO) mice were infused with Ang II to induce AAA. **a** PNU decreased the levels of NLRP3, cleaved caspase-1, GSDMD and N-GSDMD in aortas from ApoE^−/−^ mice. *n* = 6 mice per group, data were shown as means ± SD, **P < 0.01 vs. CTRL; ^##^*P* < 0.01 vs. AAA. **b** α7nAChR deficiency promoted NLRP3, cleaved caspase-1, GSDMD and N-GSDMD expression in aortas from mice with Ang II infusion. *n* = 8 mice per group, data were shown as means ± SD, *P < 0.01, **P < 0.01 vs. WT+Ang II. **c** The expressions of Cleaved caspase-11 in aortas from ApoE^−/−^ mice, or WT and KO mice with Ang II infusion. PNU decreased the levels of Cleaved caspase-11 in aortas from ApoE^−/−^ mice. *n* = 6 mice per group, data were shown as means ± SD, **P < 0.01 vs. CTRL; ^##^*P* < 0.01 vs. AAA. α7nAChR deficiency promoted Cleaved caspase-11 expression in aortas from mice with Ang II infusion. *n* = 8 mice per group, data were shown as means ± SD, *P < 0.01 vs. WT+Ang II.

As above mentioned, NLRP3 inflammasome was activated and GSDMD expression was increased in AAA models of ApoE^−/−^ mice (Fig. 1). It was well-accepted that activation of NLRP3 inflammasome could trigger auto-activation of caspase-1 [31], GSDMD could be cleaved by activated caspase-1, and the N-GSDMD was responsible for executing plasma membrane permeabilization to trigger pyroptosis [18]. Thus, the expressions of NLRP3 inflammasome and GSDMD in AAA were assessed. It was found that activating α7nAChR decreased the protein levels of NLRP3, GSDMD, cleaved caspase-1 and N-GSDMD in AAA models from ApoE^−/−^ mice (Fig. 5a). Meanwhile, α7nAChR deficiency promoted these proteins’ expression with Ang II infusion (Fig. 5b). These suggested that activating α7nAChR could inhibit cell pyroptosis through NLRP3/caspase-1/GSDMD pathway in AAA. In addition, the caspase-11-dependent non-classical pyroptosis in AAA was also detected. Compared with control, it was found that cleaved caspase-11 was significantly increased in AAA tissues from Ang II-infused ApoE^−/−^ mice, and activating α7nAChR partly prevented this change. α7nAChR deficiency further promoted the increase of cleaved caspase-11 by Ang II infusion in contrast to WT mice (Fig. 5c).

### Activating α7nAChR inhibited oxidative stress, NLRP3 activation and pyroptosis in MOVAS by TNF-α

**Fig. 6:**
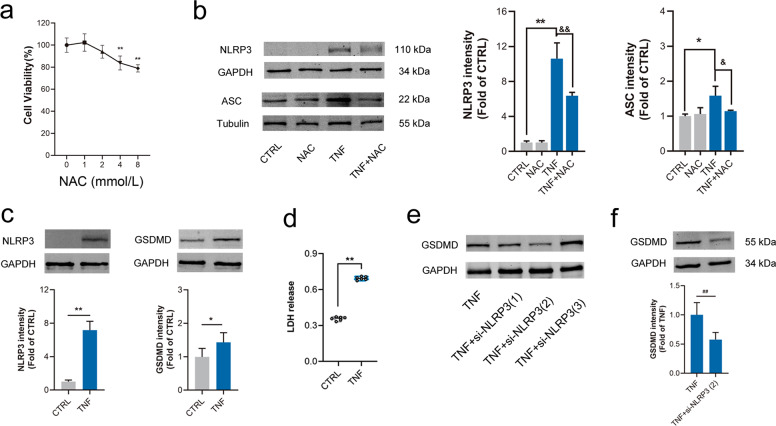
Knockdown NLRP3 inhibited GSDMD expression and LDH release in MOVAS by TNF-α. Mouse aortic vascular smooth muscle cells (MOVAS) were treated with TNF-α for 24 h. **a** Different concentrations of N-acetylcysteine (NAC, a ROS scavenger) on cell viability. *n* = 6 per group, data were shown as means ± SD. **P < 0.01 vs. CTRL (0 mmol/L). **b** The increased expressions of NLRP3 and ASC by TNF-α were inhibited by NAC (2 mmol/L). *n* = 4 per group, data were shown as means ± SD. *P < 0.05, **P < 0.01 vs. CTRL; ^&^*P* < 0.05, ^&&^*P* < 0.01 vs. TNF. **c**, **d** NLRP3 and GSDMD expression, and LDH release (*OD* at 490 nm) were increased in MOVAS cells by TNF-α. *n* = 6 per group, data were shown as means ± SD or each value, *P < 0.05, **P < 0.01 vs. CTRL. **e**, **f** NLRP3 knockdown inhibited GSDMD expression in MOVAS cells treated with TNF-α. *n* = 6 per group, data were shown as means ± SD. ^##^*P* < 0.01 vs. TNF-α.

NLRP3 inflammasome is composed of NLRP3, ASC and pro-caspase-1. It was well-accepted that NLRP3 inflammasome could be activated by ROS [31–33]. Here, MOVAS cells were treated with TNF-α in vitro and pretreated with NAC (a ROS scavenger) to confirm whether reducing ROS production could inhibit NLRP3 activation. In total, 2 mmol/L NAC was chosen in the following experiment. It was found that expressions of NLRP3 and ASC were increased in MOVAS treated by TNF-α and pretreatment with NAC partially prevented these changes (Fig. 6a, b). These supported that NLRP3 inflammasome could be activated by ROS. In addition, LDH release and GSDMD expression as well as NLRP3 expression were markedly increased in MOVAS with TNF-α stimulation (Fig. 6c, d). Designed si-NLRP3(2) was found to be the most efficient siRNA in down-regulating NLRP3 expression in MOVAS with TNF-α (Fig. 6e). Importantly, it was demonstrated that knockdown NLRP3 by si-NLRP3(2) could inhibit GSDMD expression (Fig. 6f). These results suggested that activation of NLRP3 inflammasome could trigger cell pyroptosis.

**Fig. 7:**
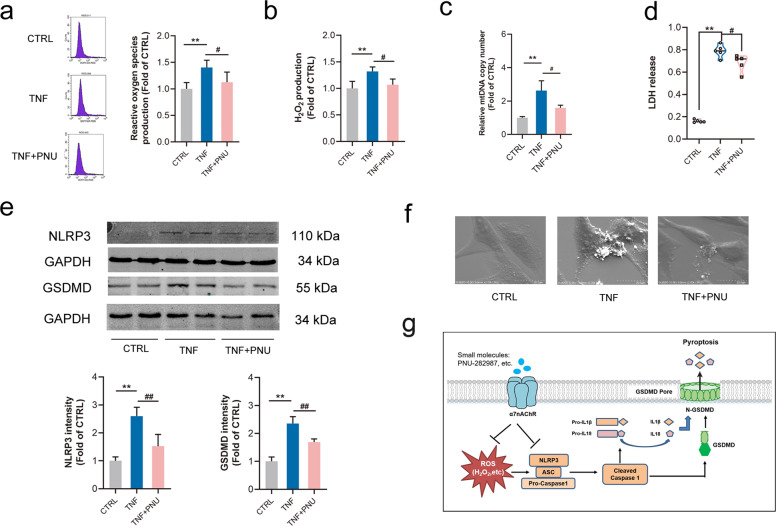
Activating α7nAChR inhibited oxidative stress, NLRP3 expression and cell pyroptosis. Mouse aortic vascular smooth muscle cells (MOVAS) were treated with TNF-α with or without pretreatment with PNU-282987 (PNU) to activate α7nAChR. **a**, **b** PNU treatment inhibited the production of ROS and H_2_O_2_ in MOVAS by TNF-α. *n* = 4 per group, data were shown as means ± SD. **P < 0.01 vs. CTRL; ^#^*P* < 0.05 vs. TNF. **c** PNU treatment reduced the relative mitochondrial DNA (mtDNA) level in MOVAS by TNF-α. *n* = 6 per group, data were shown as means ± SD. **P < 0.01 vs. CTRL; ^#^*P* < 0.05 vs. TNF-α. **d** PNU treatment reduced the release of LDH in MOVAS cells by TNF-α (*n* = 5 per group), data were shown as each value. **P < 0.01 vs. CTRL; ^#^*P* < 0.05 vs. TNF-α. **e** PNU treatment alleviated the expression of NLRP3/GSDMD in MOVAS by TNF-α. *n* = 6 per group, data were shown as means ± SD. **P < 0.01 vs. CTRL; ^##^*P* < 0.01 vs. TNF. **f** PNU treatment reduced bubble-like protrusions in MOVAS induced by TNF-α under scanning electron microscope (SEM) examination, scale bar = 20 µm. **g** The schematic representation for the mechanism of α7nAChR in AAA: Activating α7nAChR played a protective role in the pathophysiological process of AAA through inhibiting cell pyroptosis dependent on NLRP3/caspase-1/GSDMD pathway.

Evidence showed that excessive oxidative stress and apoptosis of VSMCs contribute to AAA formation [34, 35]. Under the stimulation of TNF-α, we found that productions of ROS and H_2_O_2_ were increased in MOVAS, which could be mitigated by activating α7nAChR (Fig. 7a, b). Besides, activating α7nAChR also relieved the apoptosis in MOVAS treated with TNF-α (Fig. S3). It has been reported that release of mtDNA into cytoplasm could activate NLRP3 inflammasome [36]. TNF-α stimulation increased mtDNA level, and activating α7nAChR partly prevented this change (Fig. 7c). In addition, activating α7nAChR partially down-regulated the increase of NLRP3/GSDMD expression in MOVAS with TNF-α (Fig. 7e). With a SEM examination, we demonstrated that multiple bubble-like protrusions existed in TNF-α-treated MOVAS cells and the rest of these cells kept firmly attached to the culture slide. The phenomenon was consistent with morphological features of cell pyroptosis by Chen’s work [37]. It was worthy noting that activating α7nAChR partly prevented the formation of bubble-like protrusions (Fig. 7f). Meanwhile, it was also found that LDH release was markedly high in MOVAS under TNF-α stimulation, which was reduced by PNU-282987 treatment (Fig. 7d). These results suggested that activating α7nAChR could inhibit cell pyroptosis through NLRP3 inflammasome.

## Discussion

AAA is mostly asymptomatic and can be regarded as a silent killer. Once aneurysm rupture emerges, the mortality is high. At present, there are still no therapeutic drugs to cure or prevent AAA. Therefore, to further understand the pathogenesis of AAA and seek potential targets for AAA treatment are imperative. The main findings of this study are: (1) cell pyroptosis and NLRP3 inflammasome were involved in AAA formation; (2) activating α7nAChR alleviated AAA injury and restrained inflammation; (3) α7nAChR deficiency promoted AAA formation; (4) protective effects of activating α7nAChR against cell pyroptosis in AAA were dependent on NLRP3/GSDMD pathway. In summary, we found that cell pyroptosis was involved in AAA formation and could be regulated by α7nAChR through NLRP3 inflammasome (Fig. 7g).

AAA is a disease with a more than 1.5 times localized dilation of the abdominal aorta in comparison with its initial size [38]. Ang II infusion in ApoE^−^^/−^ mice to induce AAA formation was one of the mostly used rodent model [24]. Here, we measured the maximal abdominal aortic diameter and combined with HE/EVG staining to confirm the successful establishment of AAA. It is well-accepted that cell pyroptosis is a programmed cell death in an inflammatory manner [14] and chronic inflammation in aortic wall contributes to the development of AAA [5]. Wang et al. found that cell pyroptosis in mice was dependent on activation of caspase-1, which resulted from the NLRP3 inflammasome activation [39]. Thus, activation of NLRP3 inflammasome and expression of GSDMD were detected in AAA models of ApoE^−/−^ mice. We did find that the NLRP3 inflammasome was activated as evidenced by the significantly increased protein levels of NLRP3 and cleaved caspase-1, and that the expression of cell pyroptosis effector GSDMD was dramatically increased in AAA mice.

Cholinergic anti-inflammatory pathway plays an important role in inflammatory response through α7nAChR [40]. Evidences showed that activating α7nAChR could combat inflammation and oxidative stress in a variety of cardiovascular and central nervous system diseases [41, 42], and had a protective effect after vascular injury [12]. Hence, the role of α7nAChR in AAA formation was examined. ApoE^−/−^ mice with Ang II-induced AAA was used, and PNU-282987 was injected to selectively activate α7nAChR during Ang II infusion. It was demonstrated that PNU-282987 treatment significantly reduced the maximal aortic diameter, preserved the elastin integrity, and inhibited the expression of inflammatory cytokines induced in ApoE^−/−^ mice by Ang II infusion.

To further confirm the role of α7nAChR in AAA formation, α7nAChR^−/−^ mice were used to build AAA models. In Ang II-infused mice, it was found that α7nAChR deficiency promoted inflammatory responses, accelerated the abdominal aortic dilation and displayed disruption of elastin. Consistent with the idea that VSMCs phenotype was transformed to synthetic phenotype during the development of AAA [29], it was found that activating α7nAChR significantly prevented the switch of VSMCs from contractile to synthetic phenotype in Ang II-infused ApoE^−/−^ mice, while this switch was facilitated in α7nAChR^−/−^ mice. However, duo to the limited amount of aged α7nAChR^−/−^ mice, either the AAA incidence or maximal abdominal aortic diameter only displayed an increase with no statistical significance in Ang II-infused α7nAChR^−/−^ mice when compared with the WT ones. These indicated that the role of α7nAChR in AAA was related to inflammatory responses and VSMCs phenotype switch.

It was reported that activation of NLRP3 inflammasome could trigger activation of caspase-1 [31], then GSDMD was cleaved by activated caspase-1, and subsequently the N-GSDMD initiated pyroptosis [18]. This is the so called canonical cell pyroptosis pathway. In this work, we found that activating α7nAChR significantly inhibited the expression of NLRP3, GSDMD, N-GSDMD and cleaved caspase-1 in AAA models of ApoE^−/−^ mice, while α7nAChR deficiency promoted the expression of these proteins with Ang II infusion. These suggested that activating α7nAChR could inhibit cell pyroptosis through the canonical NLRP3/caspase-1/GSDMD pathway in AAA. In view of the fact that caspase-11 was activated in AAA tissues, and activating α7nAChR partly prevented this change. We believed that caspase-11-dependent non-classical pyroptosis might be also involved in AAA formation.

Excess oxidative stress was involved in the pathogenesis of AAA [34]. ROS could activate NLRP3 inflammasome, whereas ROS scavengers could inhibit this phenomenon [43]. MOVAS cells were treated with TNF-α to simulate AAA microenvironment in vitro [44]. In this study, NLRP3 inflammasome was activated in MOVAS cells treated by TNF-α and pre-treatment with NAC (a ROS scavenger) partially prevented these changes. Cell pyroptosis as well as NLRP3 expression was also increased under TNF-α stimulation, and knockdown NLRP3 could inhibit GSDMD expression. It was well known that, activating α7nAChR could not only combat inflammation but also inhibit oxidative stress in cardiovascular and central nervous system diseases [41, 42]. Under the stimulation of TNF-α, we found that the productions of ROS and H_2_O_2_ were increased in MOVAS cells, which could be mitigated by activating α7nAChR. In addition, the increased expression of NLRP3/GSDMD in MOVAS cells with TNF-α was down-regulated by activating α7nAChR. Under SEM examination, we observed that multiple bubble-like protrusions existed in TNF-α-treated MOVAS cells, and activation of α7nAChR prevented these changes. Meanwhile, LDH (a hallmark in cell pyroptosis) release was reduced by activating α7nAChR. These suggested that activating α7nAChR could inhibit cell pyroptosis dependent on a ROS-NLRP3 pathway. In addition, activating α7nAChR could inhibit VSMCs apoptosis in vitro. PANoptosis, a form of inflammatory cell death including three major programmed cell death pathways (pyroptosis, apoptosis and necroptosis), is newly put up [45]. Therefore, the protective role of α7nAChR in AAA might be related to PANoptosis, which deserves deeper study in our future work.

In conclusion, our work demonstrated that activating α7nAChR played a protective role in AAA. Activation of α7nAChR could retard AAA formation through inhibiting cell pyroptosis dependent on NLRP3/caspase-1/GSDMD pathway. These imply that α7nAChR would be a promising target for preventing AAA formation.

## Supplementary information

Supplementary Figure 1

Supplementary Figure 2

Supplementary Figure 3

## Data Availability

The data in this article will be available on appropriate request to our corresponding or first author.
