# Supplementary figures and images for: Activating α7nAChR ameliorates abdominal aortic aneurysm through inhibiting pyroptosis mediated by NLRP3 inflammasome
Source: Acta Pharmacol Sin. 2022 Feb 25;43(10):2585–95. doi: 10.1038/s41401-022-00876-9 (PMC9525652; doi:10.1038/s41401-022-00876-9)

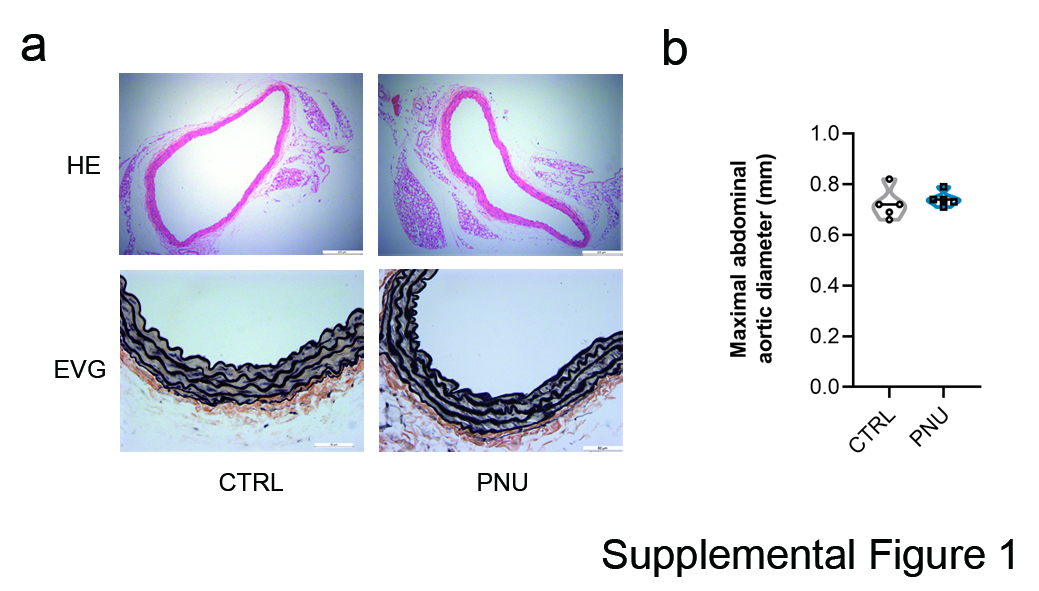

Supplement: Supplementary file 1 — Supplementary Figure 1 [file 41401_2022_876_MOESM1_ESM.tif]

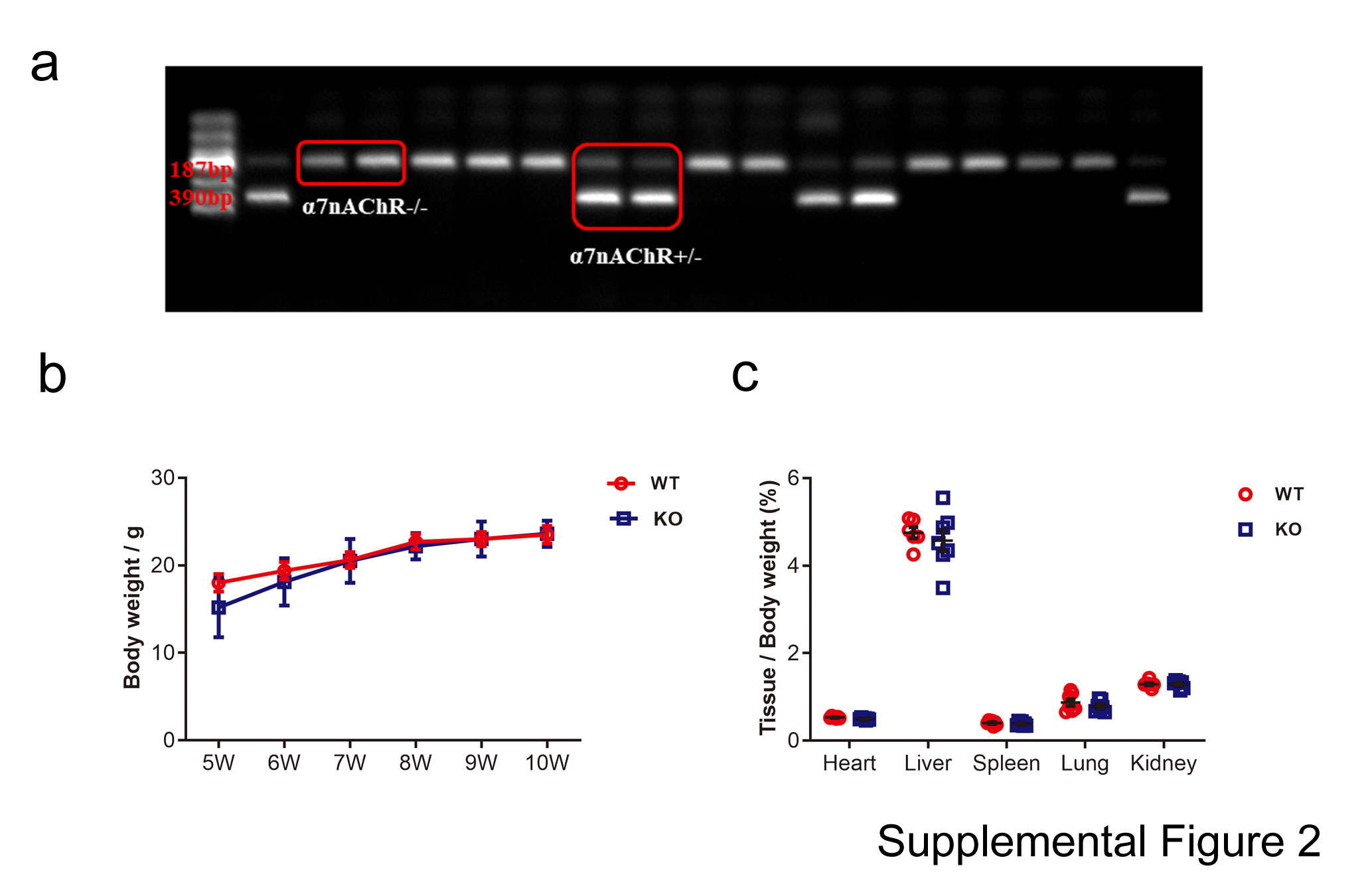

Supplement: Supplementary file 2 — Supplementary Figure 2 [file 41401_2022_876_MOESM2_ESM.tif]

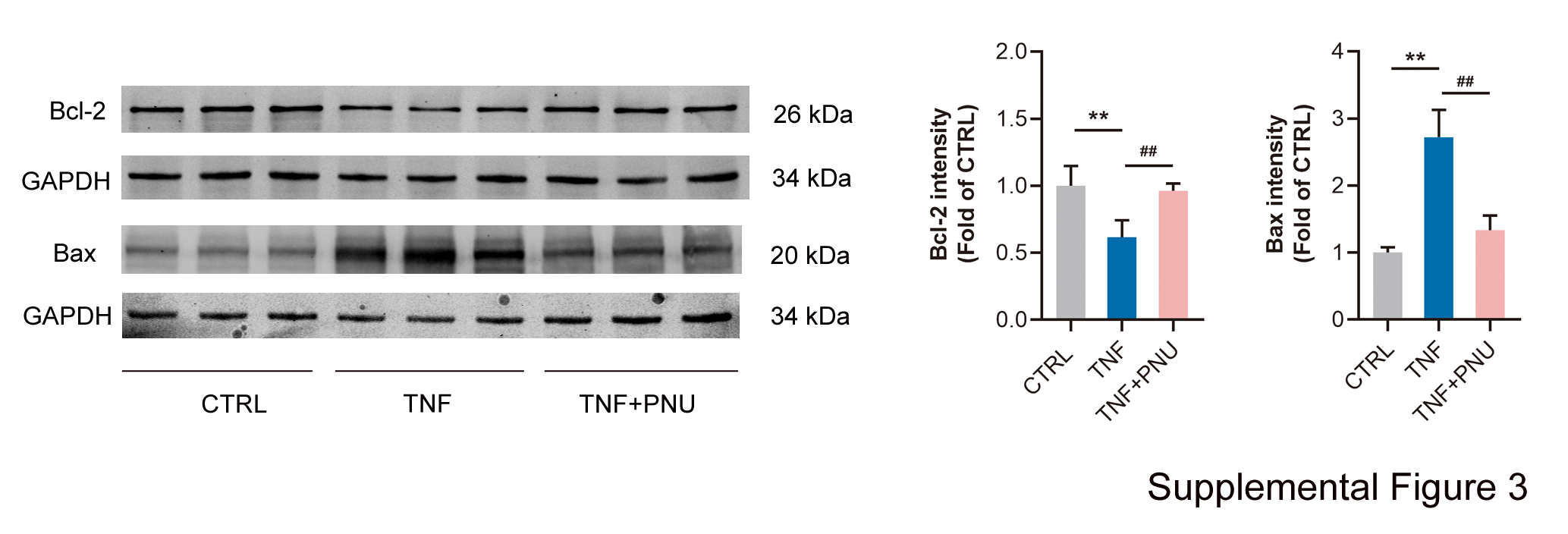

Supplement: Supplementary file 3 — Supplementary Figure 3 [file 41401_2022_876_MOESM3_ESM.tif]
